# Risk prediction tools for cancer in primary care

**DOI:** 10.1038/bjc.2015.409

**Published:** 2015-12-03

**Authors:** Juliet Usher-Smith, Jon Emery, Willie Hamilton, Simon J Griffin, Fiona M Walter

**Affiliations:** 1 The Primary Care Unit, Department of Public Health and Primary Care, University of Cambridge, Cambridge CB1 8RN, UK; 2 Faculty of Medicine, Dentistry and Health Sciences, Department of General Practice, Melbourne Medical School, The University of Melbourne, 200 Berkeley Street, Carlton, VIC 3053, Australia; 3 College House, University of Exeter Medical School, St Luke's Campus, Exeter EX1 2LU, UK

**Keywords:** primary care, cancer, risk, prediction, model, diagnosis, screening

## Abstract

Numerous risk tools are now available, which predict either current or future risk of a cancer diagnosis. In theory, these tools have the potential to improve patient outcomes through enhancing the consistency and quality of clinical decision-making, facilitating equitable and cost-effective distribution of finite resources such as screening tests or preventive interventions, and encouraging behaviour change. These potential uses have been recognised by the National Cancer Institute as an ‘area of extraordinary opportunity' and an increasing number of risk prediction models continue to be developed. The data on predictive utility (discrimination and calibration) of these models suggest that some have potential for clinical application; however, the focus on implementation and impact is much more recent and there remains considerable uncertainty about their clinical utility and how to implement them in order to maximise benefits and minimise harms such as over-medicalisation, anxiety and false reassurance. If the potential benefits of risk prediction models are to be realised in clinical practice, further validation of the underlying risk models and research to assess the acceptability, clinical impact and economic implications of incorporating them in practice are needed.

A risk prediction model aims to predict the probability or risk of a condition or event among individuals, or occasionally groups, based on a combination of known or measured characteristics. Risk prediction tools are the means by which risk prediction models, scores or algorithms are implemented in clinical practice. Numerous risk tools are now available, which predict either current or future risk of a cancer diagnosis. In theory, these tools have the potential to improve patient outcomes through enhancing the consistency and quality of clinical decision-making, facilitating equitable and cost-effective distribution of finite resources and encouraging behaviour change. These potential uses have been recognised by the National Cancer Institute as an ‘area of extraordinary opportunity' (National Cancer Institute, 2006) and an increasing number of risk prediction models continue to be developed. In the near future, risk prediction models are also likely to incorporate genomic data and could contribute to the translation of precision or personalised medicine and precision screening into clinical practice. However, there remains uncertainty about the clinical utility of risk tools and how to implement them to maximise benefits and minimise harms such as over-medicalisation, anxiety and false reassurance. In this study we provide an overview of the types of risk prediction models that exist, their potential uses, the existing evidence around their use, the challenges to implementation and the key issues for future research (see Box 1).

## What types of risk prediction models exist?

Although there is some overlap, the two main types of risk prediction model for cancer in primary care are as follows:

1. To predict the risk of prevalent but undiagnosed disease in those with symptoms.
2. To predict the risk of future incident disease in asymptomatic individuals.

Models that predict the risk of current cancer in individuals with symptoms are principally designed to guide further investigation and referral. Many have been developed for a range of cancers; in the United Kingdom the best known are the risk assessment tools (RATs) developed from case–control studies in primary care (Hamilton, 2009) and the QCancer series derived from cohorts from primary-care electronic health records (e.g., Hippisley-Cox and Coupland, 2015; Box 2).

Models that predict risk of future incident cancer are designed to identify individuals at higher risk of disease before development of symptoms. Since the first of these for cancer was described—for breast cancer in 1989—several hundred have been developed for breast and other cancers. These include at least 25 risk models for melanoma (Usher-Smith *et al*, 2014), 17 for breast cancer (Meads *et al*, 2012), 4 for lung cancer (Li *et al*, 2015), 127 for prostate cancer (Louie *et al*, 2014) and 9 for colorectal cancer (Win *et al*, 2012). Examples of those that have been externally validated for each of these cancers are given in Table 1.

## How are risk prediction models developed and evaluated?

Most are developed by applying multivariate statistical methods, usually logistic or Cox regression modelling, to data from epidemiological studies. Ideally, these are prospective cohort studies. Many of the well-known risk prediction models, however, have been developed using case–control designs either with concurrent data collection or retrospective risk factor information from electronic health records. A number of well-known models, including the Disease Risk Index developed by the Harvard School of Public Health (Colditz *et al*, 2000), have also used systematic reviews of existing studies and expert opinion.

When evaluating risk models, there are two main aspects to consider. The first is how well the model predicts the relevant outcome in the population of interest (the predictive performance) (Steyerberg *et al*, 2013; Collins *et al*, 2015). This is assessed by estimating the following: the discrimination or ability to rank individuals (e.g., sensitivity, specificity and area under the receiver operating characteristic curve (AUROC)); the calibration or ability to predict the absolute level of risk (e.g., calibration plots of observed *vs* predicted risk and the Hosmer–Lemeshow *χ^2^*-test); and the model fit or whether the model predicts disease better than chance alone (e.g., the Bayes information criterion). Ideally, risk models are developed in one population-based data set and externally validated in a second independent population-based data set, because predictive utility tends to be overestimated when models are tested in the same population in which they were developed (Collins *et al*, 2015). However, if this is not possible, re-sampling methods such as bootstrapping can be used to assess possible optimism in model performance: these methods are preferable to splitting the data into development and validation samples (Steyerberg *et al*, 2013). Over-optimism (or spectrum effect or bias) also occurs when the frequency of the outcome is inflated in development or validation samples, for example, in case–control studies in which the frequency of cancer may be 50%. This must be taken into account when considering application of a risk model to a different population.

The second aspect is whether the use of the risk tool influences clinician or patient behaviour and patient outcomes. This is addressed through implementation studies, ideally randomised controlled trials (RCTs), in which clinical and patient behaviour, patient outcomes and cost effectiveness can be assessed.

## How can the information derived from risk prediction models be used?

Once developed and evaluated, these models can be incorporated into risk prediction tools and provided on paper, as mouse mats or flipcharts, or as electronic tools, either integrated into clinical computer systems or as standalone/web-based electronic tools such as the Disease Risk Index developed by the Harvard School of Public Health (available at http://www.diseaseriskindex.harvard.edu). The output can be presented either as absolute or relative risk, rank or peer comparison, with more sophisticated tools presenting risk in a variety of formats along with the potential impact of risk-reducing interventions.

For patients with symptoms, these tools can then be used to help guide investigation and referral. For example, working in collaboration with Macmillan Cancer Support, BMJ Informatica has developed the electronic Cancer Decision Support Tool, which integrates both the RATs and QCancer models (Box 1) within general practice computer systems to provide three functions for GPs:

1. Prompts during consultations if patients have a risk of ⩾2% (adjustable) for lung, colorectal, pancreatic, ovarian or oesophago-gastric cancer, using information added as Read-codes in the past;
2. A series of ‘symptom checkers' for patients in whom GPs have identified symptoms suggestive of cancer, which enable them to enter additional symptomatic information and update the cancer risk estimates;
3. A risk stratification tool intended for use separately from consultations. Working as an audit tool, it allows practices to generate lists of all registered patients in whom a risk score can be calculated and sorts them by cancer type and risk category.

By stratifying the population into different risk levels, risk prediction tools for asymptomatic individuals have the potential not only to identify people for tailored cancer screening, behaviour change programmes and preventive treatment but also to allocate finite preventive resources more efficiently than by age and gender alone. They may also provide opportunities to motivate behaviour change among both healthcare professionals and patients, including discussions around lifestyle factors, uptake of cancer screening and potential chemo-prophylactic options, such as the use of aspirin in those at higher risk of colorectal cancer or hormone modification for breast cancer. Even for well-established cardiovascular risk tools, however, there is little evidence that simply providing patients with a number (Brindle *et al*, 2006) or genetic risk (Marteau *et al*, 2010) leads to significant changes in habitual and environmentally cued behaviours such as diet, smoking, physical activity and alcohol intake; whether providing risk information on future cancer risk will have greater effects is as yet unknown.

### What current evidence is there for risk prediction tools for cancer?

#### Tools for predicting the risk of prevalent undiagnosed cancer in symptomatic individuals

Many of the risk models for symptomatic individuals have been validated in separate populations. These include several of the QCancer risk models (e.g., Collins and Altman, 2013) and the colorectal cancer RAT (Marshall *et al*, 2011), as well as other well-known models such as the Selvachandran model for colorectal cancer (Selvachandran *et al*, 2002). These show that the models have good discrimination with AUROCs between 0.79 and 0.95, and sensitivities of 46.0–61.3 with a specificity of 95%.

By comparison, to our knowledge, there have been no published RCTs of implementation. Several feasibility studies of the QCancer or RAT models have been reported. In one, the clinical utility of two RATs (colorectal and lung) was assessed in 165 practices (Hamilton *et al*, 2013). Paper, mouse mat and desktop easel forms were provided for a 6-month period. During this time there was an increase in cancer diagnostic activity, urgent referrals and cancer diagnoses when compared with the previous 6 months. However, as this was not a trial, it is not possible to say whether these changes were due to the use of the RATs or whether they were clinically appropriate. An embedded qualitative study showed that the majority of GPs found the RATs useful in consultations and their use heightened GPs' awareness of potential cancer symptoms, reminded and alerted them to potential cancer risks and affected their thresholds (Green *et al*, 2014). Another qualitative study explored GP views following implementation of the same two RATs (colorectal and lung) in an electronic format (eRATs) (Dikomitis *et al*, 2015). This also showed that the tools raised awareness of potential cancer symptoms among GPs and affected their referral thresholds. However, there was concern about ‘prompt fatigue' whereby GPs became inured to (or irritated by) recurrent prompts, the reliance of the tool on Read codes—which are used very differently by different GPs—and the medico-legal implications of having access to a list of patients with increased risk and not acting on it.

#### Tools for predicting the risk of future incident cancer in asymptomatic individuals

Considering the number and range of risk models for predicting risk of cancer in asymptomatic individuals, few have been validated in external populations; in recent systematic reviews, only 2 out of 25 risk models for melanoma (Usher-Smith *et al*, 2014), 6 out of 17 risk models for breast cancer (Meads *et al*, 2012) and 6 out of 9 for colorectal cancer (Win *et al*, 2012) have been validated in external populations, and only 6 of the 127 models for prostate cancer have been validated in ⩾5 external populations with a reported C-statistic (Louie *et al*, 2014).

The performance, and therefore potential utility, of these models varies, in part because of different selected cut-offs for high-risk groups, but several have C-statistics over 0.7 (Table 1). As with the tools for symptomatic individuals, few of these risk tools have been subject to RCTs, to assess their clinical impact (Marteau *et al*, 2010; Walker *et al*, 2015). Your Disease Risk (YDR), based on the Harvard risk model, has been evaluated as part of a decision aid for colorectal cancer screening (Schroy *et al*, 2012). The group that received the decision aid alone had higher colorectal screening rates compared with either the control group or the group that received YDR plus the decision aid. The other large primary-care trials of risk tools for prevention have focused on family history as the principal risk factor. The GRAIDS Trial was a cluster RCT in the United Kingdom examining a web-based family history RAT for breast, ovarian and colorectal cancer (Emery *et al*, 2007). Risk was assessed using risk-assessment guidelines; participants were also provided with numerical estimates for breast cancer risk using the Claus model (Claus *et al*, 1991). Practices that received the GRAIDS software showed significant increases in referral rates and more appropriate referrals to cancer genetics services compared with those that received education and the paper guidelines. However, because of limited specificity of the risk-assessment guidelines, the GRAIDS software resulted in over-referral of people with a family history of colorectal cancer. The Family Healthware Impact Trial in the United States also tested a web-based risk assessment tool aimed for use by consumers. It assessed risk of common cancers, heart disease and type 2 diabetes using family history risk heuristics giving tailored health preventive messages (Ruffin *et al*, 2011). Those receiving the intervention were significantly more likely to increase their self-reported physical activity and intake of fruit and vegetables to recommended levels than the control group, who received standardised health messages. No differences in cancer screening rates between groups were observed, although rates at baseline were relatively high in both groups.

### What are the main challenges to implementation of risk prediction tools for cancer in primary care?

#### Choosing which risk model to use

The choice of risk model is of particular importance when used to predict future risk of disease in asymptomatic individuals. Some models include personal information, such as naevi count, dietary factors or genetic information, requiring some form of initial data collection, whereas others include only data routinely collected during clinical care, such as BMI and smoking status, and thus can be implemented with little additional resource. Although some are targeted at the general population, others include results from screening tests (such as faecal occult blood tests) and so are more applicable to a stratified risk-assessment programme. The sensitivity and specificity of the models also vary. For melanoma, for example, risk models range from those in which 50% of the population would be classified as high risk and 80% of melanomas would be detected from that high-risk group, to those in which only 20% would be identified as high risk and only 50% of cases would be detected (Usher-Smith *et al*, 2014). The choice for a given setting and health system is likely to be driven by practical and financial constraints, and the relative benefits, harms and costs of missed and over-diagnoses.

#### Choosing when and where risk should be predicted

When considering implementing risk tools for asymptomatic individuals, the challenge is to balance the benefits and harms, and demonstrate cost effectiveness. Systematic application of a risk tool to an entire population could be used to identify populations at increased risk of a cancer, who could then be offered tailored screening and primary preventive advice. It is likely to be that the use of risk prediction tools would identify those at higher risk with greater discrimination and accuracy than current simple guidelines, although recent UK guidelines were based in part on data within risk tools. However, any potential reduction in cancer incidence for a small proportion of the population needs to be balanced against possible net harms (anxiety and false reassurance) among the majority, as well as the cost of implementation and additional health care use by ‘medicalised' or ‘overdiagnosed' members of the population. As research advances into possible chemo-preventive agents such as aspirin, to reduce future risk of colorectal cancer, the potential side effects of medication and the risks of over-medicalisation need to be considered as well.

#### Understanding and overcoming barriers to use

For the tools designed for symptomatic individuals the main challenges are finding ways to make the tools accessible for clinicians and presenting risk information in an understandable format. Recent research has shown that clinicians may interpret symptoms inconsistently (Chiang *et al*, 2015), leading to inaccurate and unreliable cancer risk assessment, and although GPs were able to make sense of the purpose of the tool, they found the tool difficult to introduce into the workflow of the consultation. They may also be reluctant to use the tools for fear of alarming their patients if the risk information is presented too explicitly (Chiang *et al*, 2015). We also know from work around the use of tools that predict future risk of cardiovascular disease that lack of time, poor knowledge or understanding of the tools, the perception that clinical judgement is as good as or better than risk tools, uncertainty about how to account for risk factors perceived to be important but not included in the tools and poor computer software all form barriers to use (Van Steenkiste *et al*, 2004; Müller-Riemenschneider *et al*, 2010).

#### Communicating the risk

The format in which risk information is presented is a key aspect of risk assessment tools, as it affects both clinicians' use of the tool and patients' understanding and perception of risk. A recent study with GPs and simulated consultations using a tool that implemented QCancer suggested that GPs may prefer traffic-light colour coding of risks (red, amber and green) with secondary access to the numeric risks, if needed, rather than absolute numeric risks or being presented with diagnostic guidance as the primary output (Chiang *et al*, 2015). It is likely to be that GPs will differ in their preferences; furthermore, these may change over time and with different patients.

It is also not clear how best to present this risk information to patients. From the field of cardiovascular disease we know that numerical presentation of risk—as opposed to simple risk categories—and timeframes <10 years lead to more accurate risk perceptions. Conversely, presenting relative risk reductions maximises acceptance of treatment and ‘heart age' appears to increase intention to change behaviour (Waldron *et al*, 2011). Research is needed to see whether this is the same for cancer risk and to assess the impact of recent developments; these include expected frequency trees (Kurz-Milcke *et al*, 2008) that could potentially be of benefit when there are concerns about potential overscreening in people at average cancer risk.

#### Deciding where to set the thresholds for intervention

This is perhaps the biggest challenge for all cancer risk tools. With the current drive for earlier diagnosis of cancer in primary care, it is tempting to choose a low threshold for tools for symptomatic patients so that few individuals with cancer are missed. However, as always, there is a trade-off between sensitivity and specificity: although cancer risk prediction tools have the potential to enable better case selection for screening, preventive programmes and investigation for suspected cancer, they can also lead to overinvestigation, overdiagnosis and unnecessary harmful treatment, in particular for asymptomatic screened populations. Although their use can potentially make cancer screening more cost-effective (Chowdhury *et al*, 2013), all screening programmes cause harm and the selection of cut-offs at which to change the nature or frequency of screening will have financial and resource implications.

## Conclusions

Following in the footsteps of cardiovascular risk scores, numerous tools are now being made available in primary care to estimate, communicate and monitor risk of current undiagnosed and future incident cancer. The data on predictive utility (discrimination and calibration) of these models suggest that some have potential for clinical application. However, the focus on implementation and impact is much more recent, with considerable uncertainty about their clinical utility and how best to implement them in primary care, in order to maximise benefits and minimise harms. To ensure we make the most of this ‘area of extraordinary opportunity', future research therefore needs to focus on the following: wider validation of the underlying risk models; assessment of the utility, including patient and professional acceptability, of incorporating them into risk tools in practice; impact studies addressing whether their use affects behaviour of either patients or practitioners; and studies modelling the population level impact and economic implications of widespread use.

## Figures and Tables

**Table 1:** Examples of risk prediction models for asymptomatic individuals that have been validated in different populations

	**Summary of performance in validation studies**	
**Risk model**	**Discrimination (AUROC, 95% CI)**	**Calibration (O/E, 95% CI)**
**Breast (Meads *et al*, 2012)**		
Colditz	0.63 (0.63–0.64)	1.01 (0.94–1.09)
Gail 2	0.63 (0.59–0.67)	0.95 (0.88–1.01)
Rosner and Colditz	0.57 (0.55–0.59)	0.96 (0.92–1.02)
Tyrer and Cusick	0.762 (0.70–0.82)^a^	1.09 (0.85–1.41)^a^
**Lung (Li *et al*, 2015)**		
Bach	0.81 (0.76–0.86)	0.88 (0.72–1.08)
Sptiz	0.78 (0.73–0.83)	3.75 (3.06–4.60)
LLP	0.79 (0.73–0.83)	1.12 (0.92–1.37)
PLCO_M2012_^b^	0.81 (0.76–0.86)	1.03 (0.87–1.23)
**Colorectal (Win *et al*, 2012)**		
Harvard Cancer Risk Index	Men: 0.71 (0.68–0.74) Women: 0.67 (0.64–0.70)	—
Imperiale	0.74 (SD=0.06)	—
Freedman	Men: 0.61 (0.60–0.62) Women: 0.61 (0.59–0.62)	Men: 0.99 (0.95–1.04) Women: 1.05 (0.98–1.11)
Ma	0.64 (0.61–0.67)	1.09 (0.98–1.23)
**Prostate (Louie *et al*, 2014)**		
Prostataclass	0.79 (0.75–0.84)	—
Finne	0.74 (0.70–0.77)	—
Karakiewcz	0.74 (0.69–0.80)	—
Prostate Cancer Prevention Trial	0.66 (0.63–0.68)	—
Chun	0.76 (0.72–0.79)	—
ERSPC RC3^c^	0.79 (0.77–0.81)	—
**Melanoma (Usher-Smith *et al*, 2014)**		
Fortes	0.79 (0.70–0.86)	—
Williams	0.70 (0.64–0.77)	

Abbreviations: AUROC=area under receiver operator characteristic curve; CI=confidence interval; LLP, liverpool lung project; O/E=ratio of observed to expected events.

a In a high-risk sample.

b The 2012 version of the prostate, lung, colorectal, and ovarian cancer screening trial model.

c European Randomized Study of Screening for Prostate Cancer Risk Calculator 3.

## Box 1. Sources and selection criteria

To prepare this review we searched Medline, Embase, reference lists of major reviews and personal records using the terms ‘cancer', ‘risk model' or ‘risk prediction' and ‘primary care' or site-specific cancer terms. We focused on systematic reviews, meta-analyses, randomised controlled trials and observational studies in primary care.

## Box 2. Details of the RATs and QCancer series risk prediction tools for symptomatic individuals

**The Risk Assessment Tools**

- Designed to be used in symptomatic populations presenting to primary care.
- Developed from case–control studies in UK primary care.
- So far, tools for 14 separate cancer sites have been published (colorectal, oesophageal, lung, ovarian, kidney, bladder, pancreas, breast, uterine, brain, prostate, Hodgkin lymphoma, non-Hodgkin lymphoma and multiple myeloma).
- Provide risk estimates for patients with single symptoms of possible cancer, pairs of symptoms and repeat attendances with the same symptoms.

**The QCancer series**

- Hybrid models that can be used both in symptomatic and asymptomatic populations.
- Developed in the QRESEARCH database, a large database comprising over 12 million anonymised health records from 602 general practices throughout the United Kingdom using the EMIS (Egton Medical Information Systems) computer system.
- Six models have been published for symptomatic populations (for colorectal, gastro-oesophageal, lung, renal, pancreatic and ovarian cancer), plus models predicting risk for multiple cancers for males and females.
- Provide estimates of absolute risk of any cancer with a breakdown of type of cancer based on both risk factors such as age, gender and family history, which increase the likelihood of cancer, and risk markers such as haemoptysis or weight loss, which are features, usually symptoms, suggesting that cancer is already present.
- Available on line at www.qcancer.org
